# dp53 Restrains Ectopic Neural Stem Cell Formation in the *Drosophila* Brain in a Non-Apoptotic Mechanism Involving Archipelago and Cyclin E

**DOI:** 10.1371/journal.pone.0028098

**Published:** 2011-11-28

**Authors:** Yingshi Ouyang, Yan Song, Bingwei Lu

**Affiliations:** Department of Pathology, Stanford University School of Medicine, Stanford, California, United States of America; University of Dayton, United States of America

## Abstract

Accumulating evidence suggests that tumor-initiating stem cells or cancer stem cells (CSCs) possibly originating from normal stem cells may be the root cause of certain malignancies. How stem cell homeostasis is impaired in tumor tissues is not well understood, although certain tumor suppressors have been implicated. In this study, we use the *Drosophila* neural stem cells (NSCs) called neuroblasts as a model to study this process. Loss-of-function of Numb, a key cell fate determinant with well-conserved mammalian counterparts, leads to the formation of ectopic neuroblasts and a tumor phenotype in the larval brain. Overexpression of the *Drosophila* tumor suppressor p53 (dp53) was able to suppress ectopic neuroblast formation caused by *numb* loss-of-function. This occurred in a non-apoptotic manner and was independent of Dacapo, the fly counterpart of the well-characterized mammalian p53 target p21 involved in cellular senescence. The observation that dp53 affected Edu incorporation into neuroblasts led us to test the hypothesis that dp53 acts through regulation of factors involved in cell cycle progression. Our results show that the inhibitory effect of dp53 on ectopic neuroblast formation was mediated largely through its regulation of Cyclin E (Cyc E). Overexpression of Cyc E was able to abrogate dp53′s ability to rescue *numb* loss-of-function phenotypes. Increasing Cyc E levels by attenuating Archipelago (Ago), a recently identified transcriptional target of dp53 and a negative regulator of Cyc E, had similar effects. Conversely, reducing Cyc E activity by overexpressing Ago blocked ectopic neuroblast formation in *numb* mutant. Our results reveal an intimate connection between cell cycle progression and NSC self-renewal vs. differentiation control, and indicate that p53-mediated regulation of ectopic NSC self-renewal through the Ago/Cyc E axis becomes particularly important when NSC homeostasis is perturbed as in *numb* loss-of-function condition. This has important clinical implications.

## Introduction

Brain tumors constitute a deadly malignancy that affects a large human population, especially the children. Recently, it was demonstrated that the presence of tumor-initiating cells with features characteristic of normal tissue stem cells might be the root cause of cancer and account for the relapse of cancer after surgery [1], [2], [3]. It is therefore critically important to understand the origin of these brain cancer stem cells (CSCs) and the molecular and cellular mechanisms by which their aberrant behaviors contribute to the pathogenesis of brain tumors.

Recent studies on *Drosophila* neuroblasts have provided important insights into the biology of CSCs. *Drosophila* neuroblasts display extensive stem cell-like features. They divide asymmetrically to generate one daughter cell with renewed NSC fate and the other committed to differentiation. A growing number of factors, many of which are asymmetrically segregated, have been identified to establish NSC fate and maintain their homeostasis in *Drosophila*. They participate in diverse cellular processes including coordination of cell cycle with cell fate determination, connection of cell polarity with NSC self-renewal and differentiation, or modulation of transcriptional or translational regulatory networks to confer distinct daughter cell fates (for recent reviews see [4], [5], [6]). Loss-of-function of factors that are inherited preferentially by the differentiating daughter cell tends to result in dramatic increase of NSCs and consequently tumor-like overgrowth [7], [8]. Among these factors, Numb is the first identified cell fate determinant that limits neuroblast self-renewal by acting as a tumor suppressor. The revelation of mammalian Numb in tumorigenesis highlights the relevance of studies of *Drosophila* neuroblast behavior to cancer biology [9]. Numb has been intensively studied for molecular and cellular mechanisms underlying its regulation [10], [11], [12]. The function of Numb is regulated at two levels: its asymmetric localization and its activity as an antagonist of Notch signaling [11], [13], [14]. Recently, Polo kinase-mediated phosphorylation was shown to modulate Numb activity in *Drosophila* NSCs [15]. Overexpression of a mutant form of Numb mimicking the hyper-phosphorylated form (Numb-TS4D) interfered with the antagonistic effect of endogenous Numb on Notch signaling and resulted in ectopic neuroblasts, similar to that seen in *numb* mutant. In this study, we have used the *Drosophila numb* null mutant and the Numb-TS4D overexpression backgrounds to model brain tumor formation.

As NSCs divide asymmetrically to generate sibling cells with distinct fates and proliferation potentials, it has long been hypothesized that there exists intricate interplay between the cell cycle and the asymmetric cell division machineries. Indeed, partial attenuation of Cdc2 activity without blocking mitosis led to failures of asymmetric localization of cell fate determinants and the acquisition of distinct sibling cell fates during embryonic NSC divisions [16]. This result suggested that Cdc2 serves as a molecular connection between the cell cycle and asymmetric protein localization machineries. Recently, cell cycle genes, especially those involved in the control of G1/S transition, have been implicated in regulating the self-renewal, differentiation, and survival of NSCs and/or neural progenitors in mammals. For example, members of the Cyclin-dependent kinase inhibitor (CDKI) family such as p27 and p21, which promote cell cycle exit into a G_0_ state, have been demonstrated to negatively regulate the proliferation potential of NSCs and/or neural progenitors during embryonic or adult neurogenesis [17], [18], [19]. In addition, the retinoblastoma (Rb) protein, an inhibitor of G1 to S phase (G1-S) transition, inhibits neural progenitor self-renewal by repressing the expression of Hes1, one of the Notch pathway targets [20], [21]. Thus factors that prevent S phase entry can restrict the self-renewal capacity of NSCs and/or neural progenitors. Factors that promote G1-S transition may also influence NSC self-renewal. Cyc E is one such factor that activates CDK2 through direct binding [22], [23], [24], [25]. Either ectopic expression of Cyc E or inhibition of CDKI facilitated reprogramming of terminally differentiated cells into induced pluripotent stem cells (iPSCs). Conversely, extension of the G1 phase in human embryonic stem cells (hESCs) impairs their pluripotency and causes precocious differentiation [26]. These results suggest that having a shorter G1 phase of the cell cycle may be essential for maintaining stem cell pluripotency. How the cell cycle properties of stem cells are regulated, however, is not well understood.

The p53 tumor suppressor plays a prominent role in tissue homeostasis by controlling multiple cellular processes through diverse effectors [27], [28]. Inactivation of p53 function is associated with brain tumors, especially glioblastoma in humans [29], [30], [31], [32]. Mice lacking p53 have increased number of proliferating adult neural stem cells [33], [34]. Moreover, mice devoid of both p53 and PTEN exhibit an acute-onset phenotype of glioblastoma with NSCs showing enhanced self-renewal capacity [35], supporting that p53 plays an important role in the regulation of NSC homeostasis. However, the mechanisms of action of p53 and whether p53 exerts its pathophysiological role in brain tumorigenesis by acting on tumor mass cells in general or just a subset of tumor-initiating stem cell-like cells has not been clearly addressed. In mammals, a link between p53 and Numb has been established, with Numb promoting p53 stability through inhibition of the E3 ubiquitin ligase MDM2 [36]. Whether this relationship is conserved in *Drosophila* is not certain, since a clear fly homologue of MDM2 is yet to be identified. In this study, we tested whether p53 functionally interacts with Numb in regulating NSC homeostasis. We demonstrate that dp53 is able to prevent ectopic NSC formation in *numb* mutant or Numb-TS4D overexpression backgrounds, in an apparently apoptosis-independent manner. We provide evidence that the inhibitory effect of dp53 is mediated by Cyc E. Furthermore, we show that dp53 likely regulates Cyc E through its known transcriptional target Archipelago (Ago), which is recently shown to be a negative regulator of Cyc E [37], [38], [39]. These results thus establish a functional link between cell cycle progression and NSC self-renewal vs. differentiation choice, identify from the multitude of p53′s cellular functions one particular mode of p53 action that is critical for regulating stem cell behavior, and suggest new ways to manipulate NSC behavior for research or therapeutic purposes.

## Results

### Overexpression of dp53 suppresses the ectopic neuroblast formation phenotypes in *numb* mutant or Numb-TS4D overexpression backgrounds

**Figure 1:**
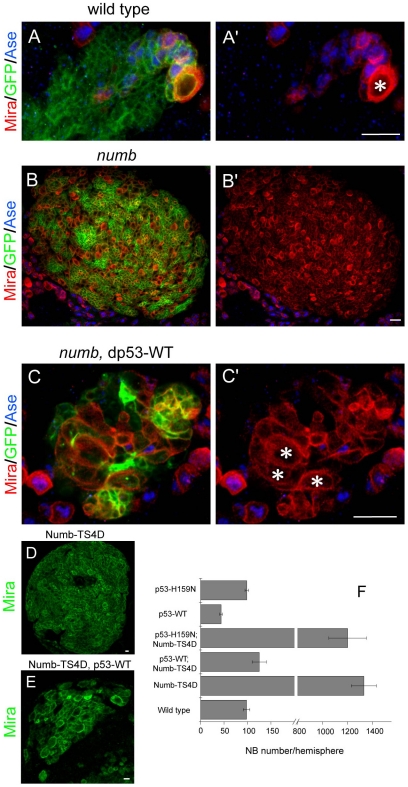
Overexpression of dp53 suppresses ectopic neuroblast formation in *numb* mutant or Numb-TS4D overexpression backgrounds. (**A–C**) Inhibition of ectopic neuroblast formation in *numb* mutant by dp53. The *UAS-dp53-WT* transgenes were introduced into *numb^15^* null mutant MARCM clones marked with GFP (green). Type II neuroblasts were identified as Miranda-positive (Mira^+^, red) and Asense-negative (Ase^-^, blue) cells with a diameter of around 10 µm and were labeled with asterisks. The smaller Mira^+^ cells are intermediate progenitors. Note that the clone in **B** and **B**' is of bigger size than that in **A** and **C** as indicated by the scale bars. The number of type II neuroblasts in dp53-expressing *numb* mutant (**C**) is much less than that of *numb^15^* mutant alone (**B**). The genotypes of each panel are *FRT40A* (**A**), *FRT40A, numb^15^* (**B**), and *FRT40A, numb^15^*, *UAS-dp53-WT; 1407-Gal4* (**C**), respectively. (**D–F**) dp53-WT (**E**), but not dp53-H159N (**F**), inhibits the ectopic neuroblast formation phenotype induced by Numb-TS4D. The corresponding transgenes were overexpressed in neuroblasts using the *1407-Gal4* driver. Neuroblasts are identified by Mira immunostaining (green). Quantification of neuroblast number is shown in **F**. Eight animals from each genotype were analyzed. Scale bars, 10 µm.

To test whether dp53 is capable of exerting a tumor suppressor function in NSC homeostasis control through functional interaction with Numb, we first overexpressed wild type dp53 (dp53-WT) in *numb* null mutant and examined the effect of dp53 on the formation of excess neuroblasts caused by Numb loss-of-function. Interestingly, enforced expression of dp53 in *numb* mutant effectively inhibited ectopic neuroblast formation (Figure 1B–C ´), indicating that dp53 is able to restrain uncontrolled neuroblast self-renewal. Numb-TS4D, a phospho-mimetic form of Numb in which 5 putative Polo phosphorylation sites have been changed to negatively charged amino acids, acted in a dominant-negative fashion and promoted ectopic neuroblast formation as well when overexpressed [15]. To test whether enforced expression of dp53 is able to suppress the ectopic neuroblast formation induced by Numb-TS4D, we co-expressed dp53-WT with Numb-TS4D in neuroblasts using the *1407-Gal4* driver. Overexpression of dp53-WT almost completely rescued the ectopic neuroblast formation phenotype in Numb-TS4D background (Figure 1D–F). In contrast, a transgene expressing dp53-H159N, a mutant form of dp53 that lacks transactivation activity, did not suppress ectopic neuroblast formation induced by Numb-TS4D (Figure 1F), indicating that the transcriptional transactivation activity of dp53 is required for its ability to restrain the formation of ectopic neuroblasts.

To test whether endogenous *dp53* normally plays a role in regulating ectopic neuroblast formation in *numb* loss-of-function background, we tested the effect of complete loss of *dp53* on a mild ectopic neuroblast formation phenotype induced by a Numb-S2D transgene [15], which represents a weak dominant-negative form of Numb (Figure S1). We did not observe obvious modification by loss of *dp53* function on the ectopic neuroblast formation induced by inactivation of Numb. It is possible that the physiological function of dp53 would only manifest itself under specific conditions (see discussion).

### The rescuing effect of dp53 overexpression on excess neuroblast formation caused by Numb loss-of-function is not due to induction of neuroblast apoptosis

**Figure 2:**
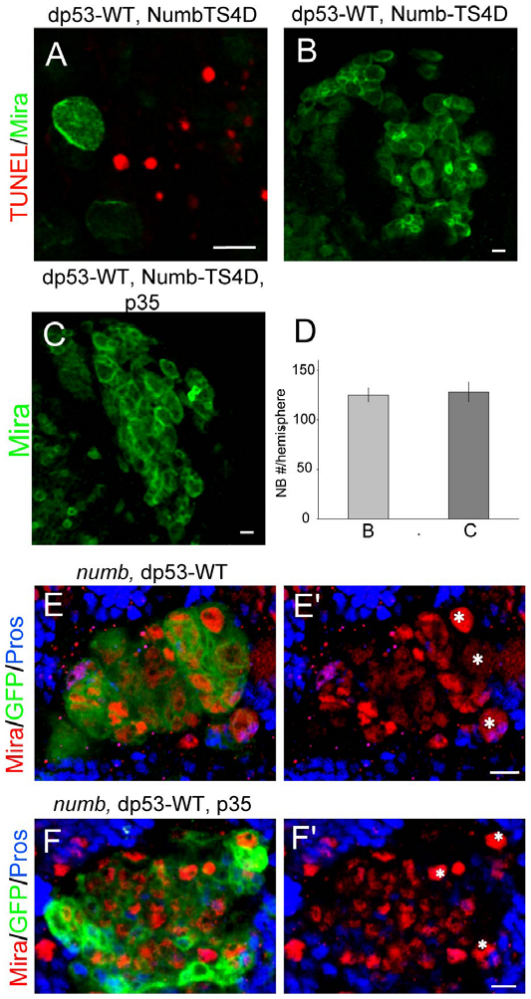
The inhibitory effect of dp53 on ectopic neuroblast formation is not due to neuroblast apoptosis. (**A**) Larval brains co-expressing Numb-TS4D and dp53-WT were analyzed for TUNEL (red) and Mira immunostaining (green). Note that TUNEL^+^ signals were not found in neuroblasts. (**B–D**) p35 is unable to block the rescuing effect of dp53 on ectopic neuroblast formation in Numb-TS4D overexpression background. The corresponding transgenes were overexpressed in neuroblasts using the *1407-Gal4* driver. Quantification of neuroblast number is shown in **D**. (**E, F**) p35 is unable to block the rescuing effect of dp53 on ectopic neuroblast formation in *numb* mutant. dp53 alone (**E, E'**) or dp53 and p35 together (**F, F'**) were overexpressed in *numb* mutant MARCM clones marked with GFP (green). Canonical neuroblasts identified as Mira^+^ cells (red) with a diameter of around 10 µm are labeled with asterisks. The smaller Mira^+^ cells are intermediate progenitors. The numbers of neuroblasts in these two genotypic backgrounds are 2.67±0.52 (**E**) and 2.83±0.41 (**F**), which are not statistically significant. The genotypes of each panel are *FRT40A, numb^15^, UAS-dp53-WT* (**E**) and *FRT40A, numb^15^*, *UAS-dp53-WT; UAS-p35* (**F**). Six animals from each genotype were analysed. Scale bars, 10 µm.

p53 is known to initiate apoptosis under stress conditions such as radiation-induced DNA damage [40], [41], [42], [43], or when overexpressed in post-mitotic neurons such as photoreceptors [43], [44]. To test whether the rescuing effect of dp53 is attributable to its induction of apoptosis in neuroblasts, we performed two experiments. First, we used TUNEL staining to detect the presence of apoptotic cells in dp53-overexpressing animals. We did observe that the overall number of TUNEL-positive nucleus was increased in dp53-overexpressing larval brains compared to control wild type brains. However, we rarely observed neuroblasts that were TUNEL-positive (Figure 2A), indicating that overexpression of dp53 did not induce neuroblast apoptosis under the conditions we used, and that most of the TUNEL-positive cells in dp53-overexpressing larval brain were post-mitotic neurons or other brain cells. The baculovirus p35 protein, an inhibitor of apoptosis, was known to be able to block dp53-induced apoptosis in the *Drosophila* eye imaginal disc [43]. We found that overexpression of p35 in neuroblasts by itself did not affect neuroblast number (Figure S2). Next we co-expressed p35 with dp53-WT in Numb-TS4D overexpression background and examined whether dp53 was still able to block the ectopic neuroblast formation effect of Numb-TS4D, when apoptosis was blocked by p35. We found that in the presence of p35, dp53 still effectively suppressed excess neuroblast formation induced by Numb-TS4D, to a similar extent as when p35 was not introduced (Figure 2B–2D). This result supports that apoptosis is unlikely to account for the rescuing effect of dp53 on the ectopic neuroblast formation induced by Numb loss-of-function. Consistently, dp53 and p35 co-expression also failed to block the ability of dp53 to suppress excess neuroblast formation in *numb* mutant MARCM clones (Figure 2E–F ´). Together, these results suggest that apoptosis is unlikely to be the main mechanism by which dp53 exerts its effect in restraining ectopic neuroblast formation when Numb function is compromised.

### The rescuing effect of dp53 on ectopic neuroblast formation caused by Numb loss-of-function is independent of Dacapo, the only *Drosophila* homologue of p21/p27

**Figure 3:**
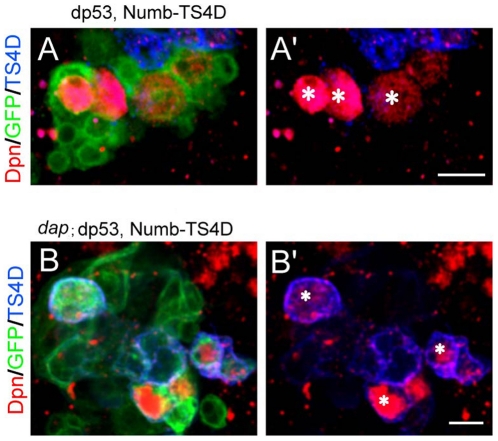
The inhibitory effect of dp53 on ectopic neuroblast formation is independent of *dap* function. (**A, A'**) Wild type neuroblast MARCM clones co-expressing Numb-TS4D and dp53. (**B, B'**) *dap* mutant neuroblast MARCM clones co-expressing Numb-TS4D and dp53. Clones were marked with GFP (green). The presence of the Numb-TS4D transgenes is shown in blue. Neuroblasts (red) are identified as Dpn^+^ cells and marked with asterisks. The numbers of neuroblasts in these two genotypic backgrounds are 2.88±0.35 (**A**) and 2.75±0.50 (**B**), which are not statistically significant. The genotypes are *FRT42D; UAS-dp53-WT, UAS-Numb-TS4D* (**A**) and *FRT42D, dap; UAS-dp53-WT, UAS-Numb-TS4D* (**B**). Eight animals from each genotype were analyzed. Scale bars, 10 µm.

p53 acts as a tumor suppressor by regulating multiple targets, including those involved in cell cycle progression [28]. Among the diverse downstream effectors of mammalian p53 that have been implicated in controlling cell cycle progression, p21 is a primary target that is significantly induced by p53 and acts as a CDKI to stall cell proliferation and induce senescence, and it has been implicated in the regulation of NSC self-renewal [45] and in mediating the effect of p53 on the reprogramming of somatic cells to iPSCs [46], [47]. The *Drosophila* homologue of mammalian p21/p27, *dacapo* (*dap*), also plays an important role in cell cycle progression [48], [49], although it does not appear to be induced by dp53 [40]. However, a functional interaction between dp53 and Dap/p21 has been observed in the fly eye [44]. To test whether the rescuing effect of dp53 observed above might be related to Dap-mediated cell cycle arrest, we co-expressed dp53 and Numb-TS4D in *dap* mutant background. *dap* mutant alone did not alter neuroblast number in an otherwise wild type background (Figure S3). Importantly, we found that the absence of *dap* did not attenuate the ability of dp53 in suppressing the formation of ectopic neuroblasts induced by Numb-TS4D, suggesting that Dap is unlikely to be involved in the regulation of neuroblast homeostasis by dp53 in this background (Figure 3).

### Overexpression of Cyc E is able to reverse the rescuing effect of dp53 on excess neuroblast formation caused by Numb loss-of-function

**Figure 4:**
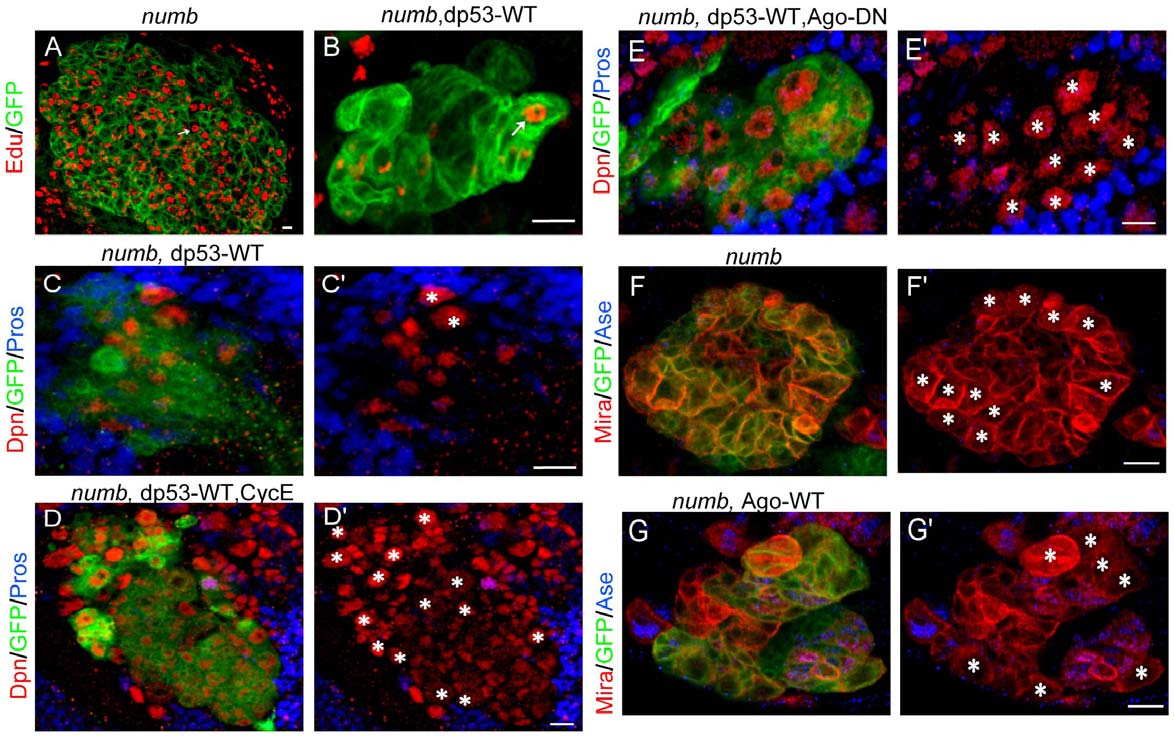
The inhibitory effect of dp53 on ectopic neuroblast formation is mediated through Cyc E. (**A, B**) The incorporation of Edu (red) into dp53-expressing *numb* mutant neuroblasts is drastically reduced compared to *numb* mutant neuroblasts without dp53 overexpression. Clones are marked with GFP (green), and cells with Edu labeling are indicated with arrows. The genotypes are *FRT40A, numb^15^* (**A**), and *FRT40A, numb^15^*, *UAS-dp53-WT* (**B**). (**C–E**) Overexpression of Cyc E (**D, D'**) or AgoΔF (**E, E'**) is able to block the rescuing effect of dp53 on ectopic neuroblast formation in *numb* mutant (**C, C'**). Clones are marked with GFP (green). Canonical neuroblasts identified as Dpn^+^ cells (red) with a diameter of around 10 µm are labeled with asterisks. The smaller Dpn^+^ cells are intermediate progenitors. The genotypes of each panel are *FRT40A, numb^15^, UAS-dp53-WT* (**C**), *FRT40A, numb^15^*, *UAS-dp53-WT; UAS-Cyc E* (**D**), and *FRT40A, numb^15^*, *UAS-dp53-WT; UAS-AgoΔF* (**E**), respectively. The differences in the number of neuroblasts in these genetic backgrounds, 2.67±0.52 (**C**, n = 7), 13.29±0.76 (**D**, n = 7), and 9.43±0.79 (**E**, n = 7), are statistically significant (*p*<0.01 in Student's *t*-test). (**F–G**) Overexpression of Ago-WT (**G, G'**) is able to rescue the ectopic neuroblast phenotype of *numb* mutant (**F, F'**). Clones were induced 48 hours ALH, different from 24 hours ALH induction shown in the other panels, and are marked with GFP (green). Mira^+^ (blue) Ase^−^ (red) cells representing type II neuroblasts are labeled with asterisks. The genotypes of each panel are *FRT40A, numb^15^*(**F**) and *FRT40A, numb^15^; UAS-Ago-WT* (**G**). The difference in the number of neuroblasts between these two genetic backgrounds, 10.5±0.76 (**F**, n = 8) and 6.75±0.46 (**G**, n = 8), is statistically significant (*p*<0.01 in Student's *t*-test). Scale bars, 10 µm.

We further explored the mechanism by which dp53 exerts its effects on neuroblast homeostasis. Extensive studies on p53 function in tumor suppression in various settings suggest that it acts through three major mechanisms: 1) Apoptosis, 2) Promotion of senescence through p21-related CDKIs, 3) Cell cycle check point control [50]. Our results have so far ruled out apoptosis and p21-related senescence as possible mechanisms. To test whether cell cycle control is involved, we conducted 5-Ethynyl-20-deoxyuridine (Edu)-labeling experiments using *numb* mutant with or without dp53 co-expression. Edu can be incorporated into newly synthesized DNA during S phase and therefore is often used as an indicator of proliferation capacity. We found that the number of Edu-positive cells was drastically decreased upon overexpression of dp53 in *numb* mutant (Figure 4A and 4B), indicating that dp53 is likely to affect G1-S transition. Cyc E is one of the key cell cycle regulators involved in G1-S transition and is considered a proliferation marker [22], [23], [24], [25]. We found that Cyc E protein level was reduced upon dp53 overexpression (Figure S4). This prompted us to hypothesize that the rescuing effect of dp53 on *numb* mutant might be caused by a negative regulation of Cyc E by dp53. To test this hypothesis, we co-expressed Cyc E and dp53 transgenes in *numb* mutant. Interestingly, we observed a significant increase of ectopic neuroblasts upon co-expression of Cyc E with dp53 in *numb* mutant, compared to overexpression of dp53 alone in *numb* mutant (Figure 4C–D ´), suggesting that exogenously provided Cyc E is able to block the inhibitory effect of dp53 on ectopic neuroblast formation in *numb* mutant. Of note, overexpression of Cyc E in a wild type background did not change neuroblast number (Figure S5A). This suggests that Cyc E is necessary but not sufficient for the induction of ectopic neuroblasts, implying the involvement of other yet to be identified components of the neuroblast self-renewal program in ectopic neuroblast formation.

### Ago acts downstream of dp53 to link dp53 with Cyc E in neuroblast homeostasis regulation

We also tried to test whether loss of Cyc E is sufficient to prevent ectopic neuroblast formation induced by Numb loss-of-function. Using two independent *Cyc E RNAi* lines, we were unable to observe discernable effect on the Numb loss-of-function phenotypes, presumably due to their inadequate knockdown of Cyc E expression. Due to the close proximity of *numb* and *cyc E* genes in the *Drosophila* genome, we have so far not been able to generate a *numb cyc E* double mutant to examine the relationship between Numb and Cyc E through a mutational approach. Therefore, we used another approach to attenuating Cyc E function. Ago is transcriptionally induced by dp53 in response to mitochondrial metabolic stress, and it has been well established that Ago can act as a negative regulator of Cyc E protein levels *in vivo* by promoting Cyc E degradation through the ubiquitin proteasome system [37], [38], [39], [51]. Such negative regulation of Cyc E by Ago appeared to be present in the *Drosophila* larval brain (Figure S6). To explore the relationship between Cyc E and dp53 in the regulation of neuroblast homeostasis, we altered endogenous Cyc E levels by genetic manipulation of Ago activity. First, we tested whether 50% reduction of *ago* gene dosage might have any effect on the ectopic neuroblast formation induced by Numb-TS4D. No effect was detected (Figure S7), presumably because 50% reduction of Ago function is not sufficient to induce Cyc E level above certain threshold level. Next we tried manipulating Ago function using AgoΔF. Overexpression of AgoΔF, a dominant-negative form of Ago lacking the F-box domain [38], is expected to increase Cyc E function by interfering with Ago-mediated Cyc E ubiquitination and degradation. Although overexpression of AgoΔF alone had no effect on neuroblast number, when AgoΔF was co-expressed with dp53 in *numb* mutant, there was a significant increase of ectopic neuroblasts compared to *numb* mutant overexpressing dp53 alone (Figure 4E–E ´), suggesting that elevation of Cyc E level through AgoΔF expression is also able to attenuate the rescuing effect of dp53.

Although overexpression of Cyc E was not sufficient to induce ectopic neuroblast formation in a wild type background, it is still possible that elevated Cyc E level is required for the formation of ectopic neuroblasts in *numb* mutant or Numb-TS4D overexpression background. To test this possibility, we examined whether overexpression of Ago-WT, which is capable of promoting Cyc E ubiquitination and degradation, would be able to rescue the ectopic neuroblast formation phenotype in *numb* mutant. Indeed, we found that while overexpression of Ago-WT alone in an otherwise wild type background had no effect on neuroblast number (Figure S5B), it reduced the number of ectopic neuroblasts by approximately 36% in *numb* mutant (Figure 4F–G ´), suggesting that Ago-WT can restrain ectopic neuroblast formation in *numb* mutant, presumably through downregulating Cyc E function.

To further assess the connection of Ago with dp53 and Numb, we performed more experiments using Numb and p53 overexpression conditions. Our results showed that either AgoΔF or Cyc E overexpression could partially suppress the neuroblast-loss phenotype induced by p53 overexpression, consistent with Ago and Cyc E being important downstream mediators of p53 function in neuroblast homeostasis regulation (Figure S8). In contrast, AgoΔF or Cyc E overexpression failed to rescue the type II neuroblast-loss phenotype induced by Numb overexpression (Figure S8). It is possible that Numb overexpression causes neuroblast loss through a mechanism distinct from p53 overexpression; alternatively, AgoΔF or Cyc E overexpression may be less effective in preventing the overexpression effect of Numb simply because they act further away from Numb than from p53 in the genetic pathway.

In addition to Cyc E, dMyc has also been shown to be a subjected to Ago-mediated degradation [52]. dMyc is potently pro-proliferative and plays important roles in cancer cell proliferation and stem cell maintenance (including iPS cells), and thus makes a reasonable candidate for mediating p53 function in regulating ectopic neuroblast formation in *numb* mutant. Surprisingly, unlike Cyc E, dMyc level did not appear to be affected by dp53 overexpression (Figure S9). Thus, while we do not exclude a possible role of dMyc in the ectopic NB formation process, it does appear that Cyc E and dMyc are differentially regulated by the p53/Ago pathway in the ectopic NB self-renewal process.

## Discussion

In this study we use the *Drosophila* neuroblast as a model system to investigate a possible role of the tumor suppressor dp53 in preventing brain tumor phenotypes caused by the formation of ectopic NSCs due to the loss of Numb function, a process that may resemble brain tumor formation from a subpopulation of CSCs in mammals. We demonstrate that overexpression of dp53 abolishes ectopic neuroblast formation in either *numb* mutant or animals overexpressing Numb-TS4D, a recently genetically engineered dominant-negative form of Numb [15]. At the mechanistic level, we show that the rescuing effect of dp53 is largely mediated by Cyc E, rather than Dap/p21, and that dp53 likely acts through the recently identified p53 target Ago to influence Cyc E. Our results underscore an intimate relationship between cell cycle progression and stem cell homeostasis and suggest that manipulating levels of cell cycle regulators such as Cyc E and Ago that can delay the cell cycle without stopping it might be therapeutically beneficial for the targeting of CSCs.

Our results indicate that dp53 acts through the Ago/Cyc E axis to regulate NSC homeostasis. Several lines of evidence are consistent with this conclusion. Firstly, we provide evidence that suppression of ectopic neuroblast formation by dp53 in *numb* mutant and Numb-TS4D-overexpression background is not due to induction of apoptosis in neuroblasts. A non-apoptotic role for dp53 in causing cellular differentiation defects has previously been observed in the fly eye [44]. Secondly, we demonstrate that the rescuing effect of dp53 is unlikely mediated by Dap, the only *Drosophila* homologue of members of the p21/p27 family in vertebrates that are transcriptionally regulated by p53 and act as inhibitors of CDKs to induce cellular senescence. Although there are evidences in mammalian systems that implicate p21/p27 family members in NSC self-renewal or maintenance control [17], [18], [19], [45], in *Drosophila* larval brain the effect of dp53 on ectopic neuroblast self-renewal in the *numb* mutant was apparently not dependent on the function of Dap. A previous study has shown that dp53 can interact with Dap/p21 to inhibit photoreceptor and cone cell differentiation in the fly eye [44], suggesting that the dp53-Dap axis might be functional in *Drosophila,* but it appears to be deployed differently in different developmental contexts. Considering the fact that overexpression of dp53 arrests neuroblasts in the G1 phase, it is likely that p53 acts on other cell cycle regulators such as Cyc E, which is critical for G1-S transition. In support of this notion, Cyc E level is reduced when dp53 is overexpressed and exogenous expression of Cyc E reversed the effect of dp53 in restraining ectopic neuroblast formation in *numb* mutant. Thirdly, our genetic interaction studies using Ago, a recently identified transcriptional target of p53 and a negatively regulator of Cyc E, also support the notion that Cyc E is a key downstream effector that mediates p53 function in NSC homeostasis control.

Interestingly, the Ago/Cyc E pathway instead of Dap/p21 was recently shown to be responsible for the activation of G1-S checkpoint in the *Drosophila* eye disc in response to metabolic stress [51], [53], suggesting that the involvement of the Ago/Cyc E pathway in mediating p53 function on the cell cycle may be more pronounced under pathophysiological conditions. Consistent with this notion, complete loss of p53 has no obvious effect on neuroblast homeostasis under normal conditions or in partial loss of Numb function background. However, it is possible that under certain unique stressful or damaging conditions that affect NSC proliferation or self-renewal vs. differentiation balance, the dp53 pathway would be activated to help maintain NSC homeostasis. Inactivation of dp53 under those conditions might compromise the homeostatic response, resulting in ectopic NSC self-renewal. This would be analogous to the observation that double-stranded breaks in DNA generated by the topoisomerase Spo11 during recombination provoked functional p53 activity, which was prolonged in cells defective for meiotic DNA repair [54]. This would also be analogous to the requirement of dp53-dependent mechanism in coordinating tissue growth in the developing wing primordium of *Drosophila*, in which slowed growth due to developmental or environmental stress in one compartment results in reduced growth and proliferation rates in adjacent cells, even though complete loss of dp53 under normal physiological conditions has no effect on the growth or proliferation of wing primordium cells [55]. Similarly, a critical role for dp53 in compensatory proliferation in *Drosophila* imaginal discs was revealed after tissue damage [56]. These studies support the notion that the diverse functions of p53 are better studied under stress or pathophysiological conditions.

Accumulating evidence supports the view that Cyc E is a limiting factor for the G1-S transition during the cell cycle. Cyc E was identified based on its ability to promote entry into S phase by direct activation of CDK2. The effects of Cyc E on cell cycle progression and cellular differentiation can be modified by its interactions with diverse components, leading to different outcomes in different cellular contexts [57], [58], [59]. For example, in terminally differentiated cells like the *Drosophila* wing and eye imaginal discs, simultaneous overexpression of Cyc E and E2F could bypass cell-cycle exit after differentiation and enable cell-cycle reentry [57]. This result suggests that preventing Cyc E-E2F from aberrant activation in terminally differentiated tissues would be critical for tissue homeostasis. In this study, exogenous expression of Cyc E alone was sufficient to bypass dp53-induced cell-cycle arrest in *numb* mutant and promote ectopic NSC formation. Overexpression of Cyc E in mammalian systems also drastically increased the efficiency of reprogramming from differentiated somatic cells to iPSCs by Oct4, Sox2, Klf4, and c-Myc [26]. It is possible that under these conditions, endogenous levels of E2F are high enough to facilitate Cyc E action, or that Cyc E can interact with other factors to achieve the same effect. Previous studies in mammals have suggested that lengthening the G1 phase is sufficient to switch neuroprogenitors from proliferative to differentiative divisions during mouse early embryonic development [60]. Thus, we propose that by downregulating Cyc E and therefore lengthening the G1 phase of the cell cycle, overexpression of dp53 promotes the differentiation of NSCs and/or intermediate progenitors in the neuroblast lineages, leading to a reduction of the number of ectopic NSCs.

**Figure 5:**
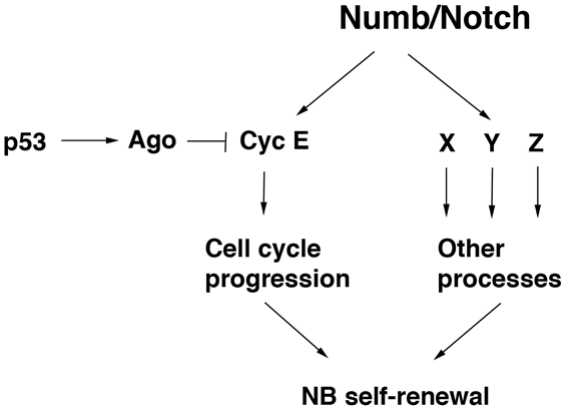
A diagram depicting the roles of Cyc E and other yet to be identified factors involved in the ectopic neuroblast formation process. A number of cellular processes such as cell cycle progression regulated by Cyc E and other processes mediated by unknown factors X, Y, Z are proposed to act coordinately to promote the self-renewal of normal or cancer stem cells. Interruption of any one of these processes, as in the case of inhibition of Cyc E expression through Ago by p53, will disrupt the self-renewal process. On the other hand, to promote ectopic neuroblast self-renewal, Cyc E and the other factors (X, Y, Z) would all have to be upregulated in a coordinated fashion. Activation of Cyc E alone will not be sufficient.

Formation of ectopic NSC due to unrestrained self-renewal is likely to be a complex process involving cell cycle progression as well as other processes such as cell growth, maintenance of undifferentiated state, and possibly stress response and genomic stability [61]. The components of the genetic program that control stem cell self-renewal have not been fully identified in any system. Our results suggest cell cycle control mediated by the p53/Ago/Cyc E pathway is one important and necessary component of the ectopic NSC self-renewal process, since disruption of this pathway has clear consequence on ectopic NB formation in *numb* mutant background. However, the p53/Ago/Cyc E pathway is clearly not the whole story, since loss of p53 or overexpression of Cyc E in an otherwise wild type background has no effect on normal NSC self-renewal. Other processes are clearly implicated by our study (Figure 5). Identifying the other critical factors that act together with Cyc E in the NSC self-renewal process will be an interesting future direction.

The dp53-mediated regulation of Cyc E through Ago in *Drosophila* NSCs revealed in this study may have important implications for understanding brain tumorigenesis in vertebrates. Loss of p53 function is frequently observed in a subset of glioblastoma that are known to contain CSCs [29], [30], [31], [32], [35]. Loss of Ago activity is also found in human tumor samples that contain CSCs [37], [38], [62]. The cancer stem cell hypothesis was initially developed in mammalian systems, where various studies support the notion that CSCs share functional features with normal stem cells, such as signaling molecules, pathways and mechanisms governing their self-renewal vs. differentiation choice. It remains poorly understood the cellular origin of CSCs and the molecular and cellular mechanisms underlying their development or maintenance. It has been proposed that CSCs could arise from 1) an expansion of normal stem cell niches, 2) normal stem cells adopting to different niches, 3) normal stem cell becoming niche-independent, or 4) differentiated progenitor cells [63]. In the *Drosophila* larval brain, CSCs can arise from the dedifferentiation of transit-amplifying progenitor cells back to stem cell-like state [64], [65]. Importantly, our study has identified the p53/Ago/Cyc E pathway as a critical axis involved in this dedifferentiation process. This dedifferentiation process may share certain similarity to reprogramming involved the production of iPSCs in mammals, which also involves p53 [50]. We speculate that elevated levels of Cyc E due to inactivation of p53 or Ago under pathophysiological conditions could have detrimental consequences on tissue homeostasis due to aberrant CSC behavior. It would be interesting to examine whether the p53-Ago-Cyc E signaling pathway characterized in this study may also play a pathological role in brain tumorigenesis in vertebrates.

## Materials and Methods

### Fly genetics

All the fly stocks and *GAL4* lines were obtained from the Bloomington *Drosophila* Stock Center, Vienna *Drosophila* RNAi Center (VDRC), or previously described in the literature. The following flies were used in this study: *UAS-dp53-WT*, *UAS-dp53-H159N*, *UAS-APC2 RNAi*, *numb^15^* [66], *UAS-AgoWT, UAS-AgoΔF* [67], *UAS-dp53 RNAi* (a gift of Dr. Andreas Bergmann), and *FRT42D, dap/cyo* [57].

To generate single neuroblast clones in MARCM analysis, virgin females of *yw hsflp; FRT40A, GAL80; tubulin-GAL4, UAS-mCD8-GFP* were crossed to males of *FRT40A, numb^15^* without or with the corresponding transgenes shown in each panel, and the larval progenies were heat-shocked at 37°C for 90 minutes, 24 hours ALH (after larval hatching) or 48 hours ALH, and further aged for 3 days at 25°C before analysis.

### Immunohistochemistry

Larval brain tissues were fixed in 4% formaldehyde according to standard procedures [11]. The primary antibodies used were: rabbit anti-Asense (1∶1000), rat anti-CycE (1∶100), guinea pig anti-Deadpan (1∶1000), chicken anti-GFP (1∶3000), mouse anti-p-Histone3 (1∶1000), rabbit anti-Miranda (1∶1000), mouse anti-Myc4A6 (1∶500), and guinea pig anti-Numb (1∶1000). Quantification of neuroblast numbers was done at 96 hrs ALH at 25°C. Larvae of each genotype were dissected and stained with neuroblast marker Miranda or Deadpan. Central brain neuroblasts can be distinguished from optic lobe neuroblasts based on their medial-superficial location, larger size and dispersed pattern.

### Western blotting

Third instar larvae overexpressing *UAS* transgenes of dp53-WT, Ago-WT with *heat shock (hs)-GAL4* driver were heat shocked at 37°C for 60 minutes and transferred to 25°C incubators for 10 hours. Then extracts were prepared from dissected larval brains and subjected to SDS-PAGE. The endogenous Cyc E levels were detected by rat anti-Cyc E antibody (a gift of Dr. Helena Richardson). Actin serves as loading control.

### TUNEL assay

ApopTag® direct fluorescein in situ apoptosis detection kit (Millipore) was used to detect apoptotic cells in larval brain in accordance with manufacturer's instructions.

### Neuroblast labeling with EdU and EdU detection

The Click-iT® EdU Alexa Fluor® 594 kit (Invitrogen) was used to assess neuroblast proliferation. In brief, the dissected larval brain was incubated with 10 µM EdU in Schneider's insect medium for 30 min. The tissues were further processed by fixation in 4% formaldehyde according to standard procedures. Incorporated EdU was detected by Alex Fluor 594 azide in accordance with manufacturer's instructions.

## Supporting Information

Figure S1 **Testing the effect of loss of dp53 on ectopic neuroblast formation induced by Numb loss of function.** Larval brain neuroblasts of *1407-Gal4>NumbS2D* (**A**), *1407-Gal4>Numb-S2D; dp53* (**B**), and *dp53* (**C**) animals were stained with Miranda (Mira). The dashed lines separate central brain neuroblasts (left) from optic lobe neuroblasts (right). Bar graph shows quantification of central brain neuroblast numbers in the different genotypes.
(TIF)

Figure S2 **Overexpression of the apoptosis inhibitor p35 has no effect on normal neuroblast number.** Larval brain neuroblasts of wild type (**A**) and *1407-Gal4>UAS-p35* (**B**) animals were stained with Miranda (Mira). The dashed lines separate central brain neuroblasts (left) from optic lobe neuroblasts (right). (**C**) Bar graph shows quantification of central brain neuroblast numbers in the two genotypes.
(TIF)

Figure S3 **Loss of** ***dap*** **has no effect on type II neuroblast self-renewal.** GFP-marked wild type (**A, A'**) and *dap* mutant (**B, B')** neuroblast MARCM clones were stained for Asense (red), GFP (green), and Mira (blue). Type II neuroblasts are Asense- Mira+. In both wild type and *dap* mutant clones, there is one and only one neuroblast/clone.
(TIF)

Figure S4 **dp53 overexpression leads to downregulation of Cyc E protein levels.** Reprehensive type II NB lineages in WT or *1407>UAS-dp53* backgrounds were circled with dashed lines. Type II NBs: yellow bracket; type I NBs: white bracket. Bar graph shows quantification of Cyc E levels in type II NBs. The difference between the two genotypes is significant (*p*<0.0001 in Student's *t* test, n = 11). Only NBs at interphase of the cell cycle were quantified.
(TIF)

Figure S5 **Control experiments showing that overexpression of Cyc E or Ago in an otherwise wild type background does not affect larval brain neuroblast number.** (**A**) wild type and *1407-Gal4>UAS-Cyc E* larval brains were stained for the neuroblast marker Dpn and differentiation marker Prospero. No difference was observed. (**B**) wild type and *1407-Gal4>UAS-Ago-WT* larval brains were stained for the neuroblast marker Dpn and differentiation marker Prospero. No difference was observed.
(TIF)

Figure S6 **Western blot analysis showing negative regulation of Cyc E protein levels by dp53 and Ago.** Control wild type (*w-*) animals or *UAS* transgenic animals overexpressing dp53 or Ago were crossed to *hs-Gal4* flies. Resulting third instar larvae were subjected to heat shock and recovery treatments and larval brain tissues were subsequently dissected out for extract preparation. Brain extracts were subjected to Western blot analysis of Cyc E and actin levels.
(TIF)

Figure S7 **Testing the effect of loss of one copy of** ***ago*** **on ectopic neuroblast formation induced by Numb-TS4D.** Larval brain neuroblasts of *1407-Gal4>Numb-TS4D* (**A**) and *1407-Gal4>Numb-TS4D; ago+/*− (**B**) animals were stained for the neuroblast marker Dpn and differentiation marker Prospero. The dashed lines separate central brain neuroblasts (right) from optic lobe neuroblasts (left). No difference was observed between the two genotypes.
(TIF)

Figure S8 **Testing the effect of AgoΔF or Cyc E overexpression on the neuroblast-loss phenotypes caused by dp53 or Numb overexpression.** Top panels: Larval brain neuroblasts of *1407-Gal4>UAS-dp53* (**A**), *1407-Gal4>UAS-dp53; UAS-Cyc E* (**B**), and *1407-Gal4>UAS-dp53; UAS-AgoΔF* (**C**) animals were stained for Miranda (Mira). The dashed lines separate central brain neuroblasts (left) from optic lobe neuroblasts (right). Bar graph shows quantification of neuroblast numbers in the different genotypes. AgoΔF and Cyc E both showed partial rescue of the neuroblast-loss induced by dp53. Bottom panels: Larval brain neuroblasts of *1407-Gal4>UAS-Numb* (**D**) and *1407-Gal4>UAS-Numb; UAS-AgoΔF* (**E**) animals were stained for Miranda (Mira). The dashed lines separate central brain neuroblasts (left) from optic lobe neuroblasts (right). Numb overexpression led to complete loss of the 8 type II neuroblasts/brain lobe, which are identifiable based on their stereotypic position and lineage composition. This phenotype was not rescued by the co-expression of AgoΔF.
(TIF)

Figure S9 **Effect of dp53 overexpression on dMyc protein level.** Top panels: Reprehensive immunostaining images of wild type and *1407-Gal4>UAS-dp53* whole brain tissues stained for F-actin (green), dMyc (red), and Pros (blue). Arrowheads point to neuroblasts that are shown at higher magnification in the bottom panels. Bottom panel: Select neuroblasts of the two genotypes shown at higher magnification. Bar graph shows quantification of dMyc levels in the two genotypes. No significant difference was detected.
(TIF)
