# Supplementary figures and images for: dp53 Restrains Ectopic Neural Stem Cell Formation in the Drosophila Brain in a Non-Apoptotic Mechanism Involving Archipelago and Cyclin E
Source: PLoS One. 2011 Nov 28;6(11):e28098. doi: 10.1371/journal.pone.0028098 (PMC3225381; doi:10.1371/journal.pone.0028098)

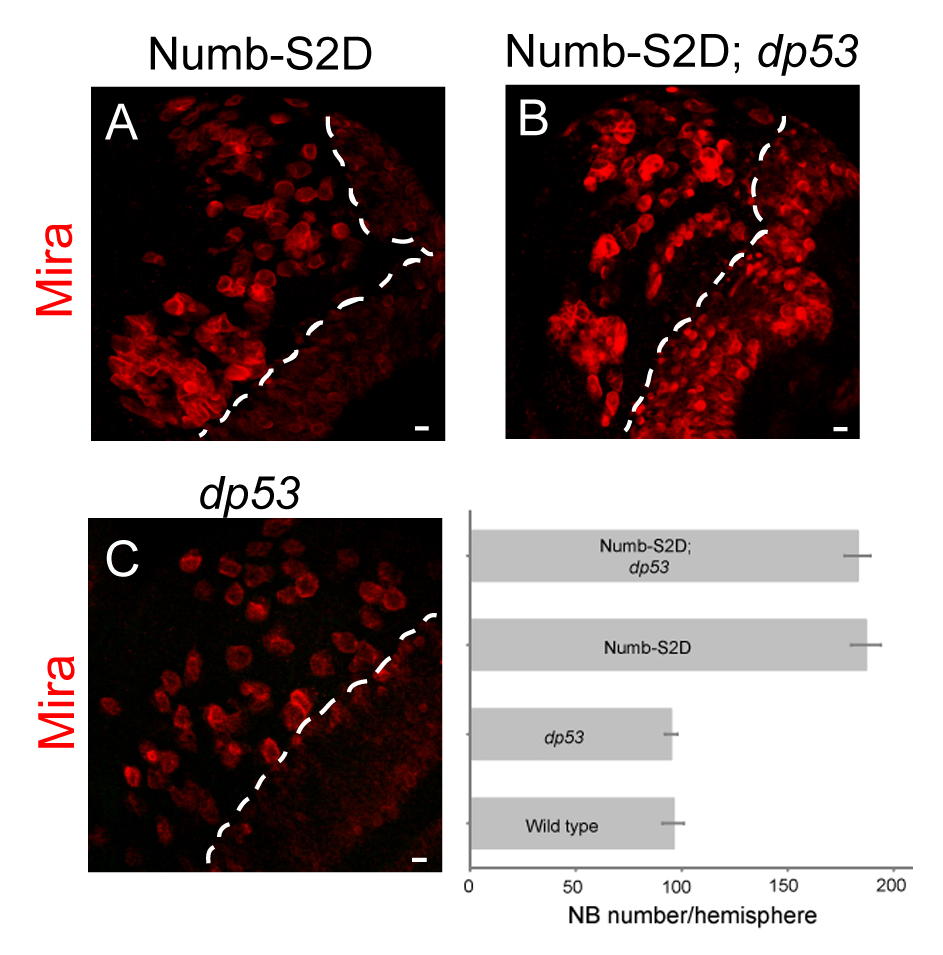

Supplement: Figure S1 — Testing the effect of loss of dp53 on ectopic neuroblast formation induced by Numb loss of function. Larval brain neuroblasts of 1407-Gal4>NumbS2D (A), 1407-Gal4>Numb-S2D; dp53 (B), and dp53 (C) animals were stained with Miranda (Mira). The dashed lines separate central brain neuroblasts (left) from optic lobe neuroblasts (right). Bar graph shows quantification of central brain neuroblast numbers in the different genotypes. (TIF) [file pone.0028098.s001.tif]

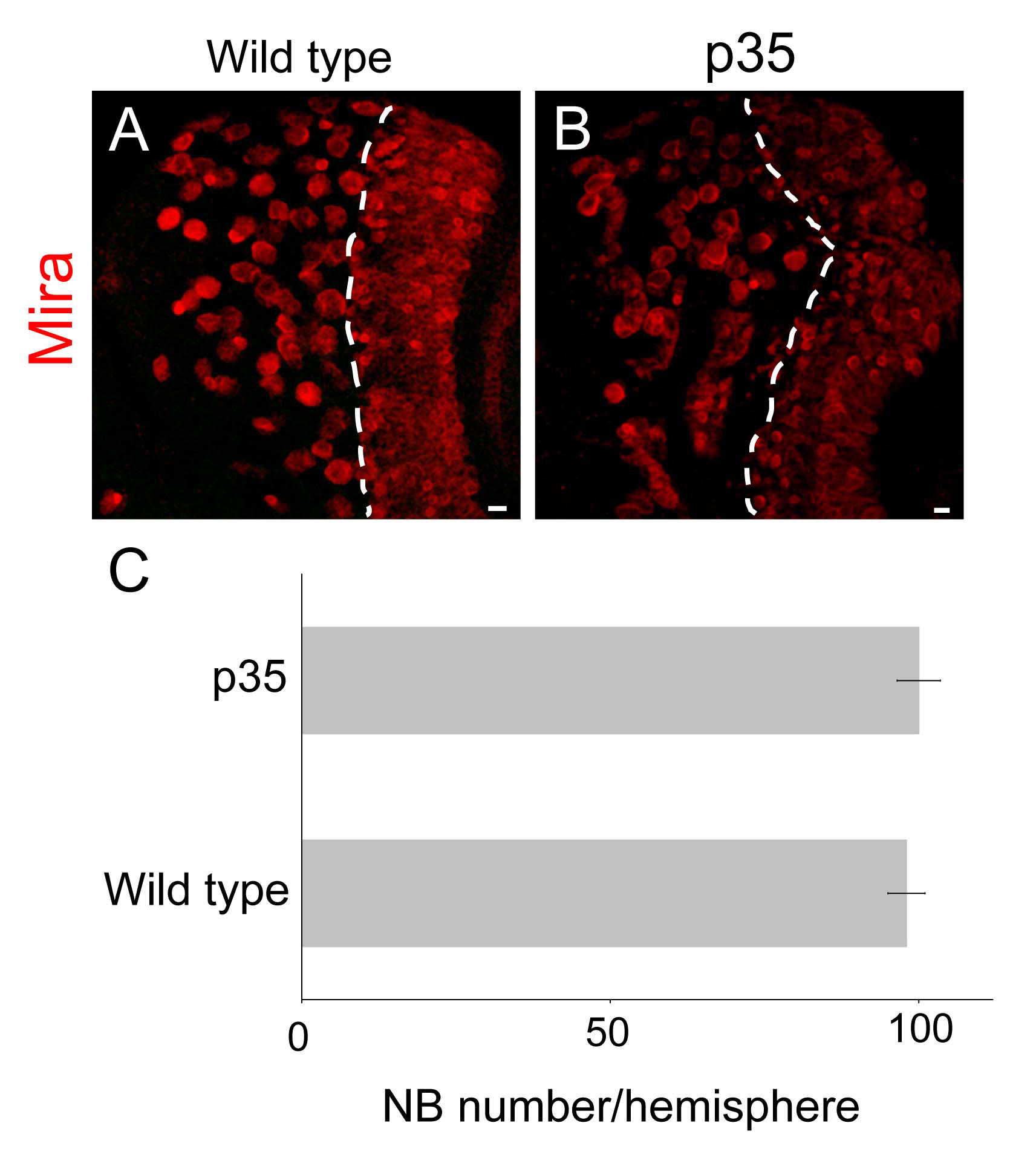

Supplement: Figure S2 — Overexpression of the apoptosis inhibitor p35 has no effect on normal neuroblast number. Larval brain neuroblasts of wild type (A) and 1407-Gal4>UAS-p35 (B) animals were stained with Miranda (Mira). The dashed lines separate central brain neuroblasts (left) from optic lobe neuroblasts (right). (C) Bar graph shows quantification of central brain neuroblast numbers in the two genotypes. (TIF) [file pone.0028098.s002.tif]

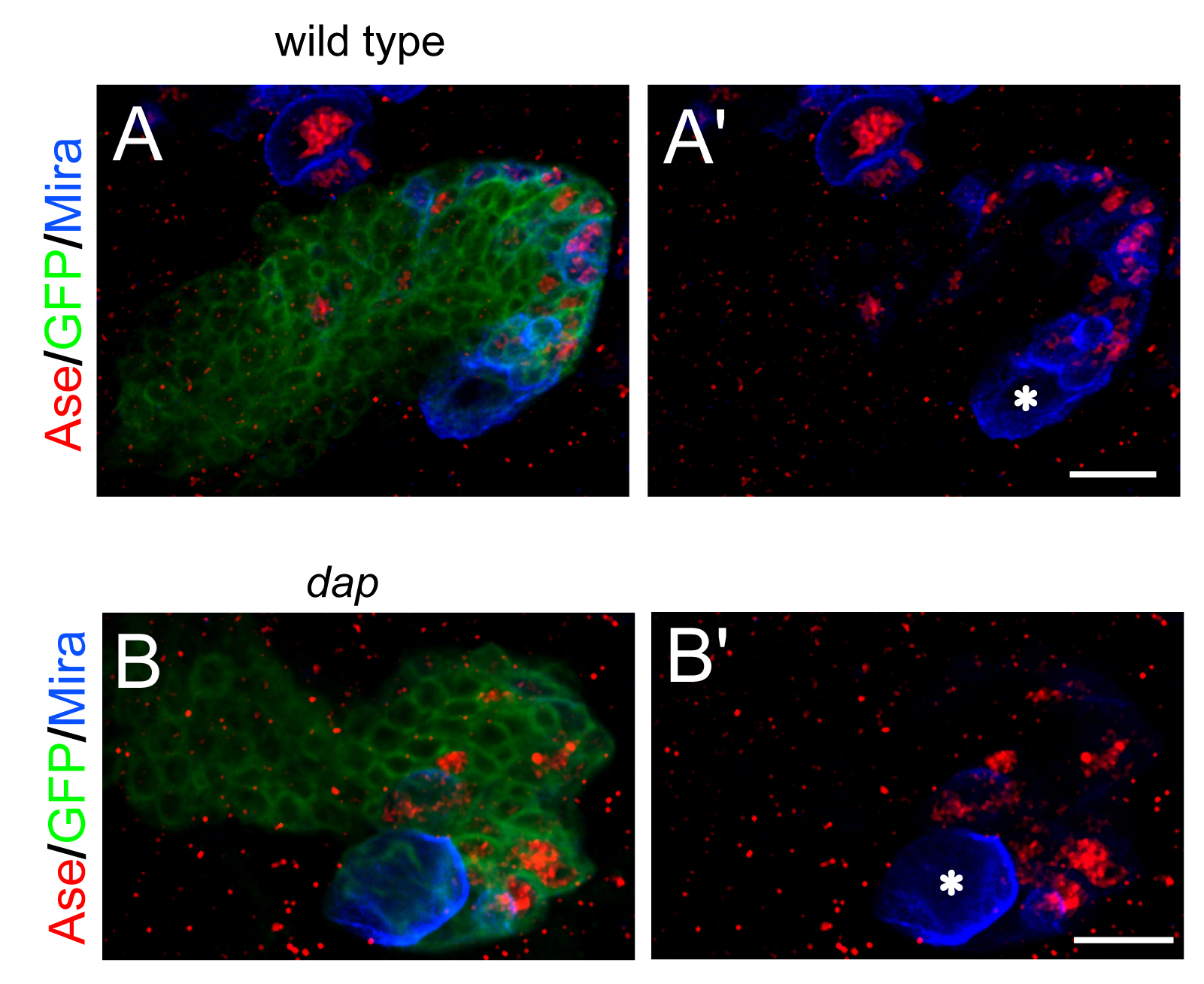

Supplement: Figure S3 — Loss of dap has no effect on type II neuroblast self-renewal. GFP-marked wild type (A, A') and dap mutant (B, B') neuroblast MARCM clones were stained for Asense (red), GFP (green), and Mira (blue). Type II neuroblasts are Asense- Mira+. In both wild type and dap mutant clones, there is one and only one neuroblast/clone. (TIF) [file pone.0028098.s003.tif]

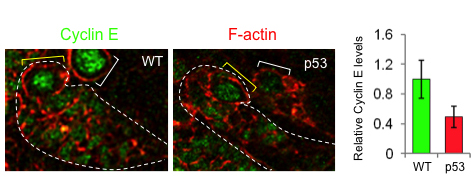

Supplement: Figure S4 — dp53 overexpression leads to downregulation of Cyc E protein levels. Reprehensive type II NB lineages in WT or 1407>UAS-dp53 backgrounds were circled with dashed lines. Type II NBs: yellow bracket; type I NBs: white bracket. Bar graph shows quantification of Cyc E levels in type II NBs. The difference between the two genotypes is significant (p<0.0001 in Student's t test, n = 11). Only NBs at interphase of the cell cycle were quantified. (TIF) [file pone.0028098.s004.tif]

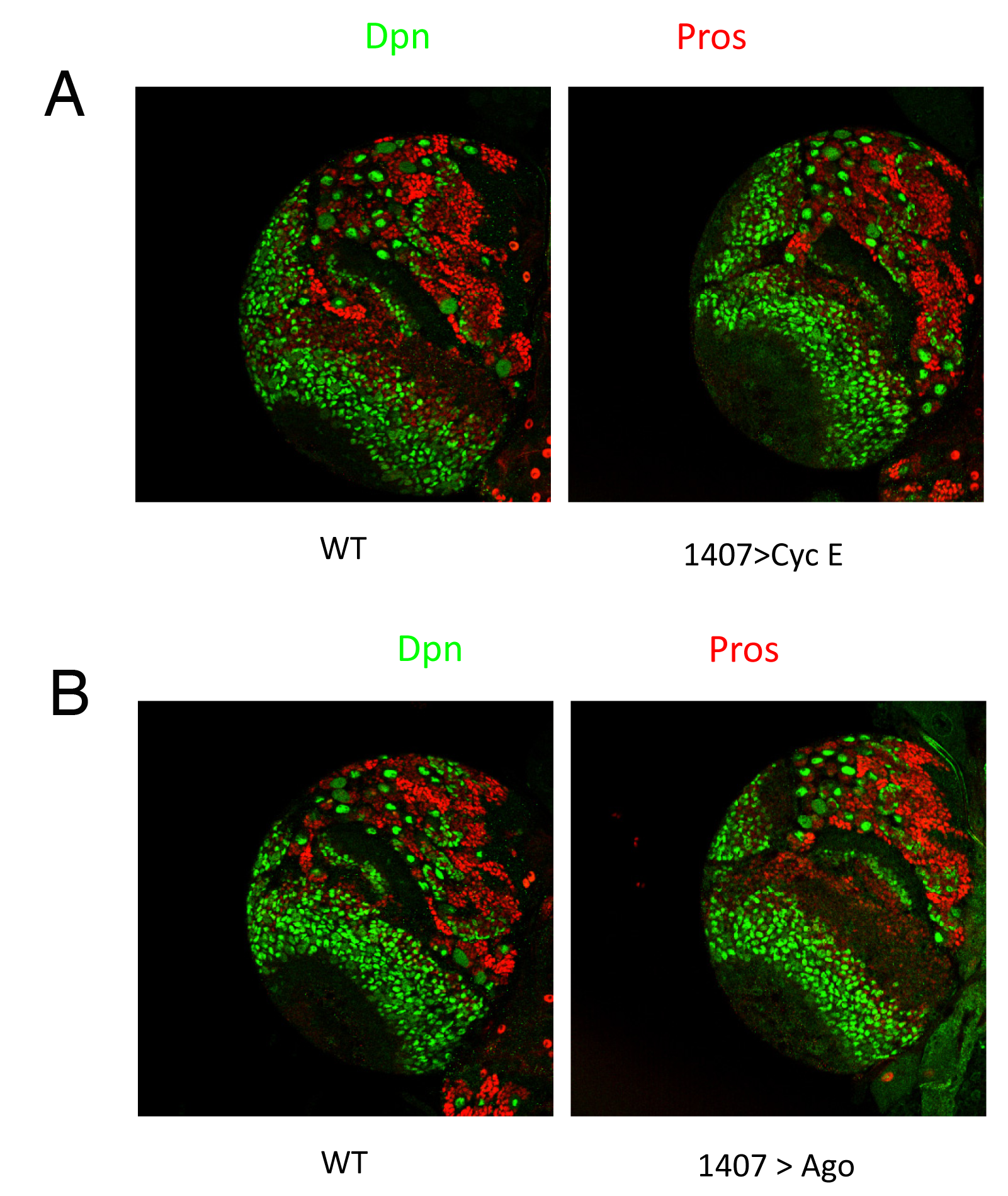

Supplement: Figure S5 — Control experiments showing that overexpression of Cyc E or Ago in an otherwise wild type background does not affect larval brain neuroblast number. (A) wild type and 1407-Gal4>UAS-Cyc E larval brains were stained for the neuroblast marker Dpn and differentiation marker Prospero. No difference was observed. (B) wild type and 1407-Gal4>UAS-Ago-WT larval brains were stained for the neuroblast marker Dpn and differentiation marker Prospero. No difference was observed. (TIF) [file pone.0028098.s005.tif]

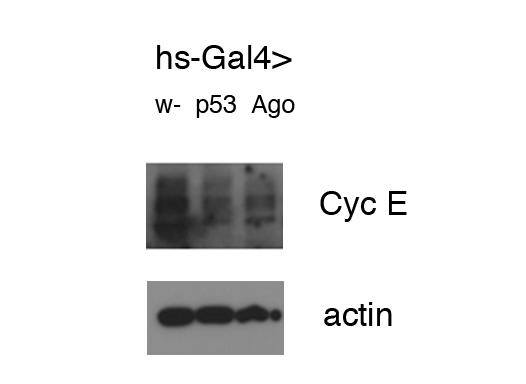

Supplement: Figure S6 — Western blot analysis showing negative regulation of Cyc E protein levels by dp53 and Ago. Control wild type (w-) animals or UAS transgenic animals overexpressing dp53 or Ago were crossed to hs-Gal4 flies. Resulting third instar larvae were subjected to heat shock and recovery treatments and larval brain tissues were subsequently dissected out for extract preparation. Brain extracts were subjected to Western blot analysis of Cyc E and actin levels. (TIF) [file pone.0028098.s006.tif]

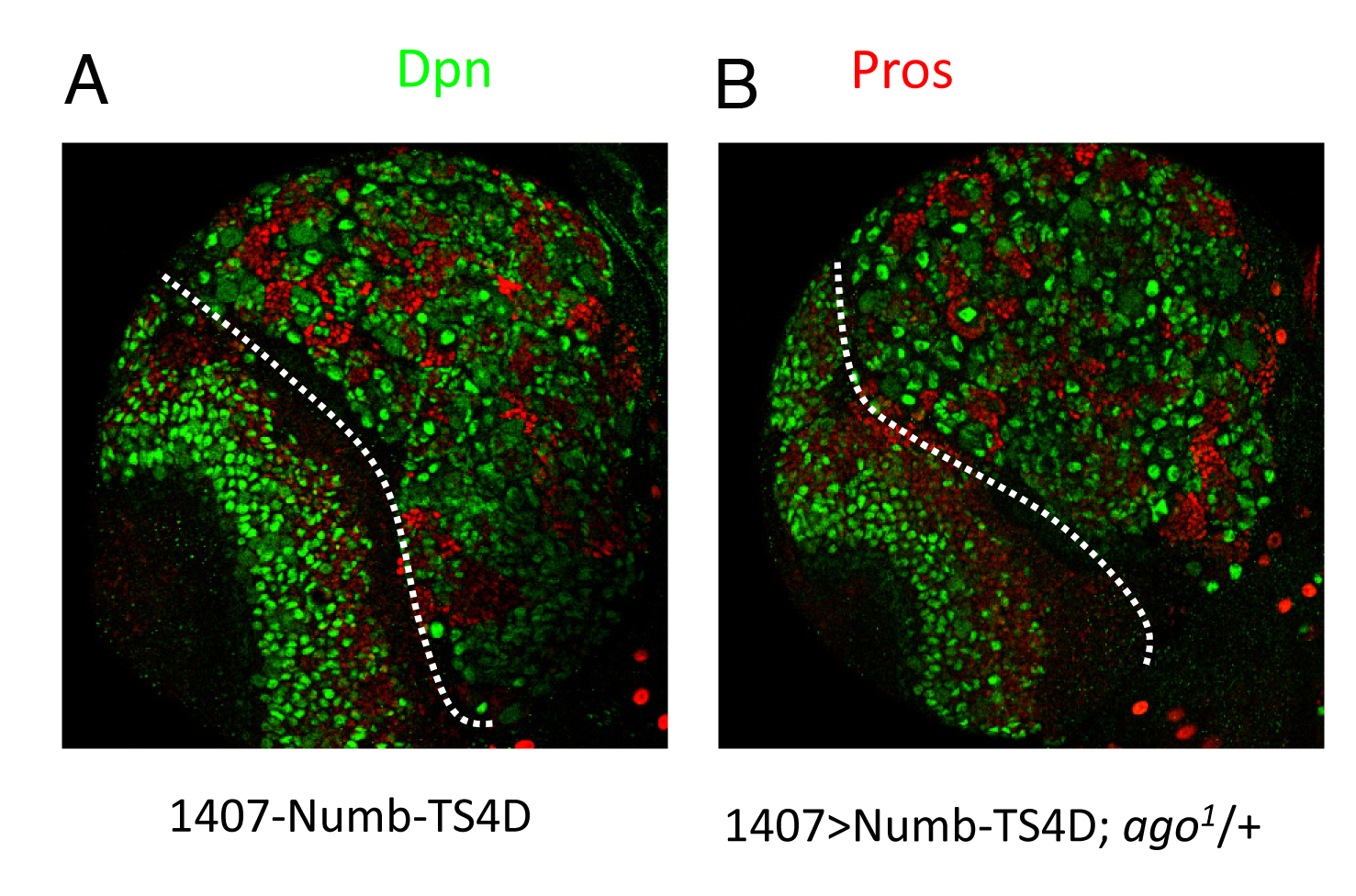

Supplement: Figure S7 — Testing the effect of loss of one copy of ago on ectopic neuroblast formation induced by Numb-TS4D. Larval brain neuroblasts of 1407-Gal4>Numb-TS4D (A) and 1407-Gal4>Numb-TS4D; ago+/− (B) animals were stained for the neuroblast marker Dpn and differentiation marker Prospero. The dashed lines separate central brain neuroblasts (right) from optic lobe neuroblasts (left). No difference was observed between the two genotypes. (TIF) [file pone.0028098.s007.tif]

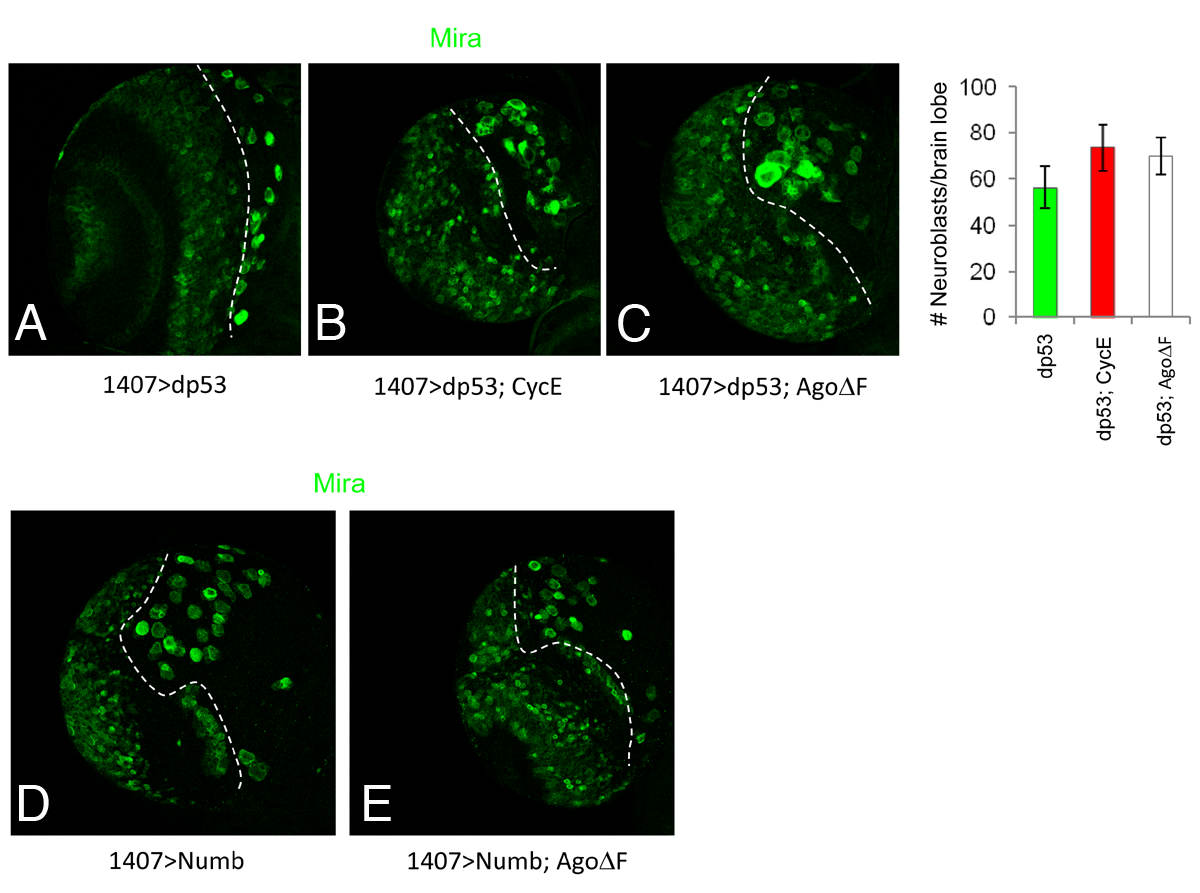

Supplement: Figure S8 — Testing the effect of AgoΔF or Cyc E overexpression on the neuroblast-loss phenotypes caused by dp53 or Numb overexpression. Top panels: Larval brain neuroblasts of 1407-Gal4>UAS-dp53 (A), 1407-Gal4>UAS-dp53; UAS-Cyc E (B), and 1407-Gal4>UAS-dp53; UAS-AgoΔF (C) animals were stained for Miranda (Mira). The dashed lines separate central brain neuroblasts (left) from optic lobe neuroblasts (right). Bar graph shows quantification of neuroblast numbers in the different genotypes. AgoΔF and Cyc E both showed partial rescue of the neuroblast-loss induced by dp53. Bottom panels: Larval brain neuroblasts of 1407-Gal4>UAS-Numb (D) and 1407-Gal4>UAS-Numb; UAS-AgoΔF (E) animals were stained for Miranda (Mira). The dashed lines separate central brain neuroblasts (left) from optic lobe neuroblasts (right). Numb overexpression led to complete loss of the 8 type II neuroblasts/brain lobe, which are identifiable based on their stereotypic position and lineage composition. This phenotype was not rescued by the co-expression of AgoΔF. (TIF) [file pone.0028098.s008.tif]

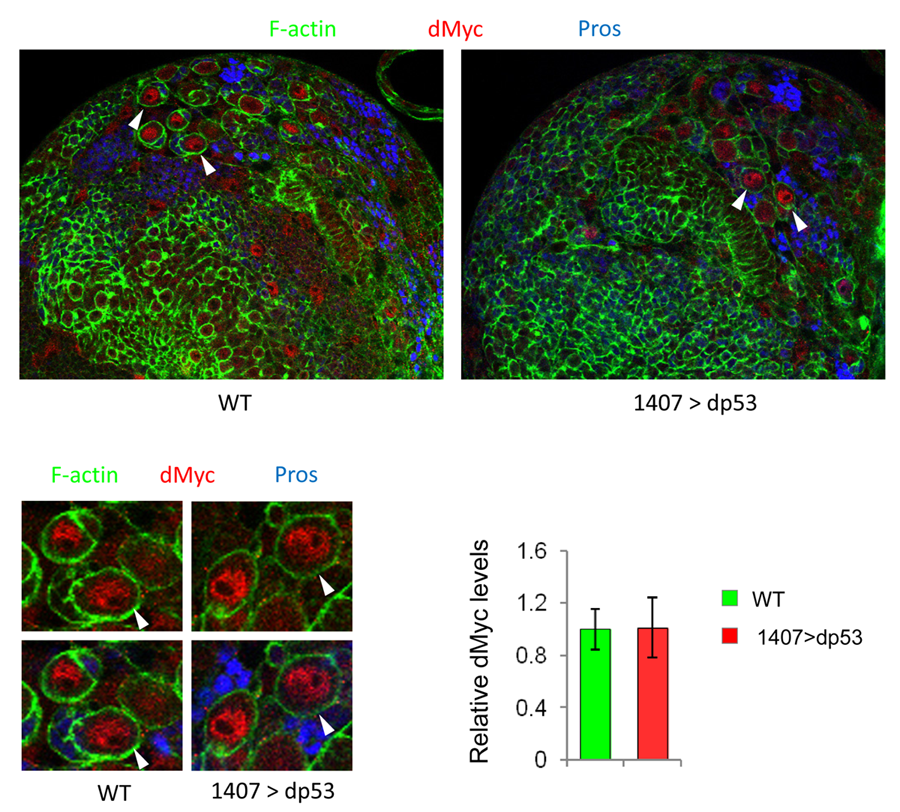

Supplement: Figure S9 — Effect of dp53 overexpression on dMyc protein level. Top panels: Reprehensive immunostaining images of wild type and 1407-Gal4>UAS-dp53 whole brain tissues stained for F-actin (green), dMyc (red), and Pros (blue). Arrowheads point to neuroblasts that are shown at higher magnification in the bottom panels. Bottom panel: Select neuroblasts of the two genotypes shown at higher magnification. Bar graph shows quantification of dMyc levels in the two genotypes. No significant difference was detected. (TIF) [file pone.0028098.s009.tif]
